# Peptides and Peptidomimetics as Potential Antiobesity Agents: Overview of Current Status

**DOI:** 10.3389/fnut.2019.00011

**Published:** 2019-02-18

**Authors:** Maushmi S. Kumar

**Affiliations:** Shobhaben Pratapbhai School of Pharmacy and Technology Management, SVKM'S Narsee Monjee Institute of Management Studies-NMIMS, Mumbai, India

**Keywords:** antiobesity, endogenous peptides, marine peptides, food peptides, peptidomimetics

## Abstract

There is a high occurrence of obesity worldwide without many new medications being approved for its treatment. Therefore, there is an urgent need to introduce new approaches for treating obesity. Bioactive peptides have been used to treat metabolic disorders- such as type-2 diabetes and obesity; while also possessing anti-oxidant, anti-inflammatory, anti-microbial, and anti-viral properties. However, the development of these peptides has taken backstage due to their size, reduced stability, poor delivery and bioavailability, fast rate of degradation etc. But with the emergence of newer techniques for multifunctional peptides, mimetics, peptide analogs, and aptamers, there is a sudden revival in this therapeutic field. An increased attention is required for development of the natural peptides from food and marine sources which can mimic the function of mediators involved in weight management to avoid obesity. Herein, the search for the structures of anti-obesity peptides was carried out in order to establish their potential for drug development in future. An extensive search for the current status of endogenous, food and marine peptides, with reference to novel and interesting experimental approaches based on peptidomimetics for controlling obesity, was performed. Apolipoprotein A-I (apoA-I), melanocortin-4 receptor (MC4R)-specific agonist, GLP-1 dual and triple agonists, neuropeptides and prolactin-releasing peptide mimetics were specifically examined for their anti-obesity role. Novel peptides, mimetics, and synthesis interventions are transpiring and might offer safer alternatives for otherwise scarcely available safe antiobesity drug. A deeper understanding of peptides and their chemistry through the use of peptide engineering can be useful to overcome the disadvantages and select best mimetics and analogs for treatment in future.

## Introduction

Obesity is an abnormal condition which involves accumulation of excessive body fat and increases the risk of associated health problems. The worldwide prevalence of obesity has almost tripled since 1975, which is an alarming and dreadful sign of impairment of human health. It increases the likelihood of diseases like type-2 diabetes, cardiovascular diseases, obstructive sleep apnea, osteoarthritis and depression (1, 2). Recently, Reilly et al. (3) pointed out at the low sensitivity of body-mass index (BMI) for classifying obesity and suggests to consider large biases for the condition. The undesirable side effects of drugs like Orlistat, phentermine/topiramate, lorcaserin, bupropion/naltrexone, liraglutide 3.0, phentermine, and diethylpropion restrict their uses and thus limit the availability of safe anti-obesity drugs (4).

**Figure 1:**
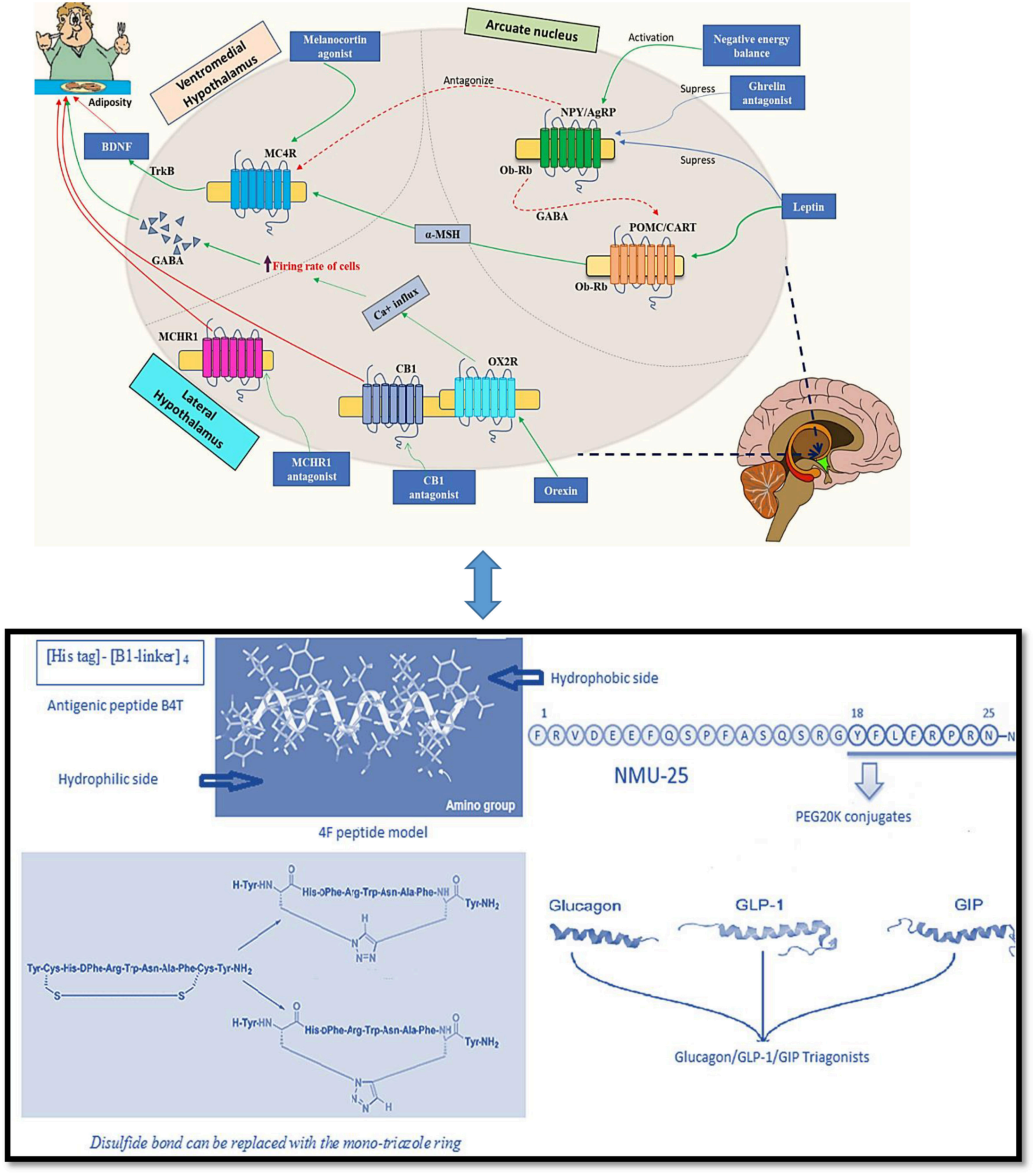
Endogenous peptides and peptidomimetics approaches for treating obesity [Adapted from Navab et al. (9), Tschop et al. (10), and Inooka et al. (11); Kim et al. (12); Tala et al. (13)] (*Indications- Green solid arrow-activation, blue solid arrow-suppression, red dashed arrow-antagonism, and red solid arrow-block*).

Endogenous as well as bioactive peptides from other sources having twenty amino acid residues exhibit anti-obesity property (5–7). Analogs for novel targets such as amylin, leptin, GLP-1 MC4R, oxyntomodulin, neuropeptide Y antagonists, cannabinoid type-1 receptor blockers, MetAP2 inhibitors, lipase inhibitors and anti-obesity vaccines are currently being studied and it is predicted that the combined use of two or more classes of drugs involving various pathways might be beneficial (8). In Figure 1 the role of major endogenous peptides, which help in regulating obesity by different pathways and exhibit combined effect is composed. α- melanocyte stimulating hormone (α-MSH) is a melanocortin 4 receptor (MC4R) agonist and is stimulated by leptin. The MC4R likewise initiates brain-derived neurotrophic factor (BDNF) through tyrosine receptor kinase B (TrkB) receptors in the ventromedial (VMH) area of the hypothalamus. BDNF, when administered centrally in db/db mice, decreased food allowance and expanded vitality consumption, exhibiting its role in controlling sustenance balance which is possibly intervened through MC4R (14, 15). α- MSH actuates MC4R at the paraventricular nucleus, which leads to a suppression in food intake and an increase in energy expenditure (16). Leptin increases BDNF production by causing an increase in the release of α-MSH from the Arcuate nucleus (Arc). It also acts on MC4R which is expressed by BDNF-producing neurons; these events demonstrate the involvement of BDNF in the control of energy expenditure and as a sequential target of leptin. The secretion of BDNF leads to its interaction with trkB situated in the paraventricular nucleus (PVN), dorsomedial hypothalamus (DMH), Arc and ventromedial hypothalamus (VMH), causing reduced food intake by stimulating the feedback mechanism. Interestingly, leptin is also responsible for causing direct activation of phosphoinositide 3-kinase (PI3K) in pro-opiomelanocortin (POMC) neurons while generating an inactivation in Agouti-related protein (AgRP) neurons (17). Neuropeptide Y (NPY)/(AgRP) neurons are inhibited by insulin and leptin and are activated in negative energy conditions. NPY/AgRP neurons estrange the melanocortin signaling while γ-amino butyric acid (GABA) discharged from NPY/AgRP neurons inhibit the POMC neurons (18, 19). Ghrelin increases food intake by actuating NPY/AgRP neurons, which results to express the ghrelin receptors and consequently adjusts the inhibitory signs from peptide YY, leptin and insulin (20).

A therapeutic focus on the treatment of obesity includes development of ghrelin receptor antagonist which can cause multiple fold increase in appetite stimulation from the gut to the mind; ghrelin receptor inverse agonists would stop essential function of the ghrelin receptor, accordingly expanding the reaction to inhibitory signals to obstruct “between-suppers” food consumption (21). Reports show that use of recombinant adiponectin in fat mice enhances fatty acid digestion along with improvement in the condition of insulin resistance. Both leptin and adiponectin are adipocytokines from adipose tissue and are noteworthy insulin-sensitizing hormones (22). Adiponectin seems to act by enacting and phosphorylating AMP-activated protein kinase (AMPK), which at that point actuates and phosphorylates acetyl CoA-carboxylase, an enzyme downstream of AMPK in adipose tissue (23). MCHR1-deficient mice are impervious to the orexigenic activities of MCH and maintains leanness with increased vitality. Administration of MCH diminishes adrenocorticotropic hormone (ACTH) level, which controls the production of glucocorticoid. An increase in glucocorticoid levels are observed in MCH-deficient mice, demonstrating the importance of MCHR1 in adrenal functions (24). Orexins perform their actions through the ventral posterior Arc where actuation of OX2R orexin receptors empowers a Na^+^/Ca^2+^ trade current in GABAergic neurons and accordingly causing depolarization and enhancing the firing rate in the cells (25). As GABA works for a powerful upgrade in feeding behavior, orexins may use this system to control craving. Another peptide oxyntomodulin reduces appetite and increases body temperature; it aids weight loss and can be administered for a longer period. A renewed interest in this peptide has led to a new and stable self-assembling nanofibril formulation development, which subsequently releases pharmacologically active peptide, having anti-obesity and anti-diabetic potential (26).

**Figure 2:**
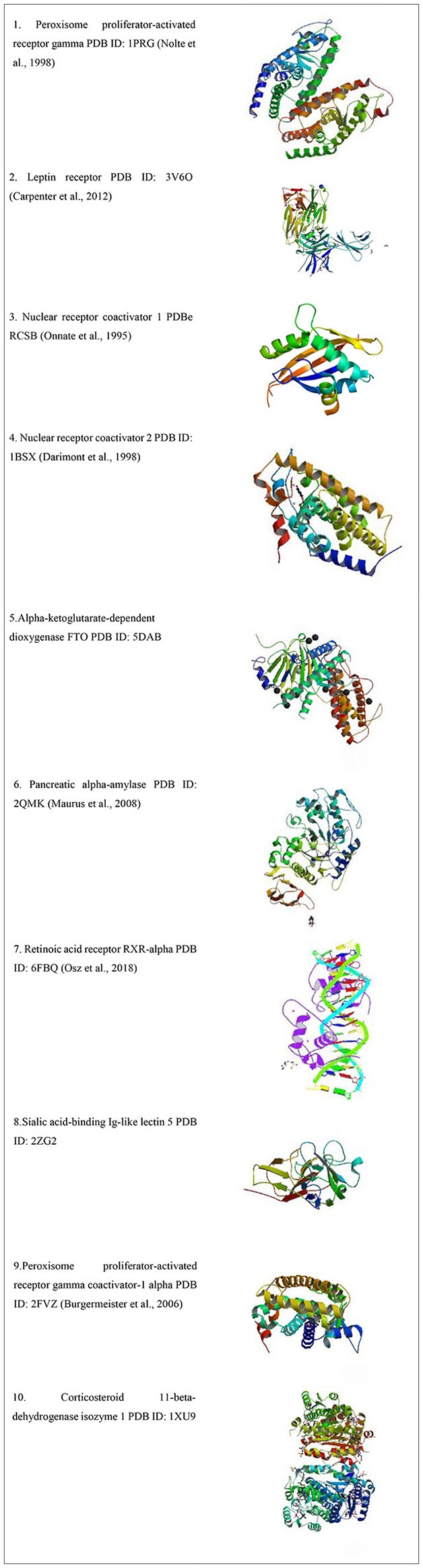
3D structures of obesity receptors from Protein Data Bank (27–33).

Protein Data Bank (PDB) was accessed for the type of available obesity receptors from *Homo sapiens*, which yielded 103 results as listed in Table 1 and the receptors are shown in Figure 2.

**Table 1:** Major receptors studied for anti-obesity activity for various ligands.

**Sr. no**.	**Receptor type**	**Studies reported**
1	Peroxisome proliferator-activated receptor gamma	53
2	Leptin receptor	1
3	Nuclear receptor coactivator 1	8
4	Nuclear receptor coactivator 2	3
5	Alpha-ketoglutarate-dependent dioxygenase FTO	16
6	Pancreatic alpha-amylase	10
7	Retinoic acid receptor RXR-alpha	4
8	Sialic acid-binding Ig-like lectin 5	3
9	Peroxisome proliferator-activated receptor gamma coactivator-1 alpha	1
10	Corticosteroid 11-beta-dehydrogenase isozyme 1	4

Herein the status of natural anti-obesity peptides from food and marine sources is updated and experimental mimetic approaches for controlling obesity is discussed in further sections.

## Peptidomimetic-Based Therapy in Obesity

Bioactive peptides face several challenges affecting their prolonged use and development, mainly due to their- chemical instability, hydrolysis, and aggregation, due to misfolding, short half-life, elimination, less permeability to cell membrane etc. However, a peptidomimetic approach by editing the naturally occurring peptides is currently being used for the development of promising drugs. They include various types of chemical modifications, L to D form isomerization, synthetic amino acid substitution, cyclization etc. (34). Researchers are targeting dipeptidyl peptidase-IV, STAT signaling, protein-protein interactions, arthritis, cardiovascular diseases, antimicrobial, immunomodulators with peptides and petidomimetics (35–41). Due to a limited number of antiobesity pharmacological drugs, chemists are now looking beyond traditional peptides and working on multifunctional peptides, peptide engineering, peptide aptamers, and peptidomimetics as modern alternatives involving newer design strategies. Aditpotide, is a peptidomimetic designed for weight loss and consists of an amino acid sequence of CysLysGlyGlyArgAlaLysAspCysGlyGlyAsp(LysLeuAlaLysLeuAlaLys)2. It reduced the body weight of the treated animals in the study by Kolonin et al. (42) and Barnhart et al. (43). In the first study, there was a rapid reduction in obesity without any adverse effects; while in the second study a remarkable reduction in white adipose tissue was observed by magnetic resonance imaging and X-ray absorptiometry. Barnhart et al. (43) showed that at experimentally determined optimal doses, monkeys from three different species displayed predictable and reversible changes in renal proximal tubule function. When engineering a new “Two-in-one” peptide, Day et al. (44) introduced a cyclic amide function in the peptide chain to make the complex more stable, and also locked a huge polyethylene glycol group to prevent its filtration from the kidney. These prudent modifications in the peptide exhibited full potency at both the glucagon and GLP receptor and caused dramatic weight loss in obese mice. Davalintide (AC2307), an amylinomimetic reduced food intake, thus decreasing body weight (45). It was also later discovered that combined administration of davalintide, an amylinomimetic, and a peptide YY (PYY) is advantageous in treating obesity (46). ValGlyPhe peptide profile was checked in obese mice and in type-2 diabetic patients. D'Amato et al. (47) conducted an experiment in a small group of patients and could conclude that the response of ValGlyPhe peptide was down regulated to glucose in both euglycemic obese patients and T2D subjects. Another combination of a synthetic peptide and a GHS-R1a antagonist compound led to the development of JMV 2959 with peptidomimetic approach using 1, 2, 4-triazole for designing ghrelin receptor ligands and investigating it for food intake and obesity (48). While it is reported that the metabolic instability can be overcome by development of d-amino acids containing small cyclic peptides, low oral bioavailability still remains a serious limitation for their pharmaceutical applications (49). Peptides and their mimetic approaches are further discussed in detail.

### Apolipoprotein Mimetic Peptides

Apolipoprotein A-I (apoA-I) is highly abundant and one of the major protein components of high-density lipoprotein (HDL). Its role of having an inverse relation in the development of obesity is well-established (50). The relation between HDL/apoA-I and autophagy has also advanced the knowledge surroudning its anti-obesity results (51). ApoA-I mimetics are reported to decrease adiposity. The anti-obesity effect of apoA-I and an apoA-I mimetic peptide D-4F were studied. The overexpression of apoA-I and treatment with the mimetic D-4F remarkably reduced white fat mass and improved insulin sensitivity moderately. ApoA-I and mimetic D-4F when used for treatment, increased uncoupling protein mRNA and proteins along with stimulated phosphorylation of AMP-activated protein kinase (AMPK) in brown adipocytes (52). Administration of another apo A-I mimetic peptide L4F in low-density lipoprotein receptor (LDLR)-null mice also influenced weight gain and total plasma cholesterol (53). ApoA-I mimetic peptide L-4F improved the metabolic profile of male ob and female mice. L4F caused a significant reduction in the visceral fat to whole body weight ratio in male mice, while causing an opposite effect in female mice; a small and significant increase in the ratio of visceral fat to body weight compared with controls, suggesting sexually dimorphic effects of L-4F (54). 4F peptide (a hydrophobic analog of 18A) is highly hydrophobic in nature due to increased phenylalanine substitutions. The increased oxidized lipid association may be due to their ATP-binding cassette transporter (ABCA1)-dependent cellular cholesterol acceptance (55). Apolipoprotein A-I mimetic peptides, synthesized from D-amino acids, were found to be stable in the circulation when administered orally to LDL receptor-null mice. The lesions reduced without any change in HDL-cholesterol level. It was partially available in circulation during the study when given by oral route to reduce atherosclerosis (9). A peptide vaccine (B4T) (an apolipoprotein B100 mimotope) has also been developed which prevents obesity condition induced by high fat diet (HFD) in wild type mice as well as in liver steatosis (Figure 1). It targeted an epitope present in ApoB100 leading to weight reduction (56).

### Melanocortin-4 Receptor Agonists

Peptide analogs of melanin concentrating hormone (MCH) with K_b_ of 0.1–0.2 μM and 4.3 nM were reported by Audinot et al. (57, 58) after the studies were carried out in rats. Bednarek et al. (59) reported another peptide as a MCH-R1 antagonist with IC_50_ value 14 nM and K_b_ of 0.9 nM. HisPheArgTrp, a tetrapeptide sequence from α-MSH and analogs were used for an antiobesity effect, due to their binding to the melanocortin-4 receptor (MC4R). It showed an increase in the metabolic stability and intestinal permeability when studies were carried out with a synthesized library of backbone cyclic peptidomimetic derivatives. Cepoi et al. (60) tested another peptide mimetic (1, 2, 3R, 4-tetrahydroisoquinoline-3-carboxylic acid) MC4R-specific agonist and its effect on inhibiting feeding behavior in mice. It decreased food intake after 4 h of intracerebroventricular (ICV) injection in mice. Peptide 1 (BL3020-1) was selective toward MC4R, had better cellular permeability in enterocytes and also increased intestinal metabolic stability. This peptide reduced food intake in mice due to the changes in backbone cyclization (61). Another antagonist ALB-127158(a) reduced food intake in DIO mice in preclinical testing and its Phase I clinical trials have been completed (62). Tala et al. (13) designed a library of 1, 2, 3-triazole bridged peptidomimetics of chimeric AGRP-melanocortin peptide. Their study concluded that the disulfide bond can be interchanged with the mono-triazole ring without affecting their functional ability at the melanocortin receptors.

### GLP-1 Dual and Triple Agonists

A glucagon-like peptide 1 (GLP-1) analog named liraglutide maintained weight loss in obese individuals at a dose of 3 mg/day (63). It is licensed by the FDA and EMA for the management of obese patients. In another approach, the synergistic effect of GLP-1 and CCK pathways were tried with a fusion peptide C2816. It was constructed with AC3174 (GLP-1R agonist) and AC170222 (CCKR1-selective agonist); C2816 reduced more body weight in obese mice as compared to the GLP-1R and CCKR1-selective agonist co-administration at a higher dose (64). Zhou et al. (65) also synthesized 24 GLP-1/glucagon receptor dual agonists. A fatty acid laurate maleimide conjugate showed good results in lowering body weight in obese mice. A triple incretin agonist GLP-1-GIP-GCG has been described as potent anti-diabetic and anti-obesity agents (66) (Figure 1).

### Neuropeptide

A short peglyated analog of a neuropeptide Neuromedin-U (Figure 1) facing short half-life led to its PEG20k conjugate which exhibited a good anti-obesity effect. At a dose of 10 and 30 nmol/kg, it reduced the body weight by 10 and 22%, respectively, in an obese mice model (11). In literature reports, a gut hormone fragment peptide YY3–36 reduced the hunger and also the intake of food in normal weight subjects (67). Catestatin (CST), a natural peptide occurring in the body also reduced body weight (68). Subsequently, a Y2R agonist was synthesized, PYY-1119(4-imidazolecarbonyl-[d Hyp24,Iva25,Pya(4)26,Cha27,36,γMeLeu28,Lys30,Aib31] ProTyrTyr (23–36) with better pharmacokinetic properties to alleviate emesis (69). Another study, in an attempt to extend the half-life, four apelin-13 analogs, namely, (Lys8GluPAL)apelin-13 amide, pGlu(Lys8GluPAL)apelin-13 amide, Lys8GluPAL(Tyr13)apelin-13, and Lys8GluPAL(Val13)apelin-13 were modified. It prolonged the half-life of native apelin-13 upto more than 24 h (70). A shorter peptide analog of gastrointestinal peptide, ProTyrTyr3–36, reduced appetite by activating the neuropeptide Y2 receptor (Y2R) thus controlling obesity and other metabolic diseases. A 14-amino acid ProTyrTyr analog, Ac-[d-Pro24, Pya (4)26, Cha27, 36, Aib28, 31, Lys30] ProTyrTyr (23–36) was administered continuously for a 2-week long study in mice, which showed high agonist and binding affinity for the Y2R. It showed a strong antiobesity effect resulting in more than 10% weight loss (71, 72).

### Prolactin-Releasing Peptide Mimetics

Prolactin-releasing peptide (PrRP) analogs have been used for development of antiobesity agents. In a recent study, PrRP showed anorectic effects when the N terminus of PrRP was attached with palmitic acid. Later palmitoylation of PrRP31 improved its bioavailability by using two linkers (γ-glutamic acid and a modified polyethylene glycol). Two-week treatment with palmitoylated analogs reduced the body weight in a diet-induced obese mouse model. It also reduced the liver weight along with leptin, triglyceride and insulin levels (73).

## Food Peptides

Soybean peptides have been used in food items extensively for their body fat-decreasing property. LeuProTyrProArg, a peptide from soybean glycinin A5A4B3 subunit and ProGlyPro are reported to have anorectic properties (74, 75). It decreased serum glycerides and cholesterol without decreasing body proteins in humans (76). Isolates from soy protein reduce the triglycerides and mRNA level of fatty acid synthase in adipose tissue in Wistar rats (77). Novel black soy peptides activated leptin-like signaling and AMP-activated protein kinase in a study for acute effects of black soy peptides on food intake and body weight in rats (78). Recently, Ashokan et al. (79) reported a peptide (ValHisValVal) from soybean responsible for stimulating lipolysis in apoptotic skeletal muscles caused by high fat diet. ValHisValVal peptide also regulated TNF-α expression which was elevated due to a high fat diet and suppressed apoptosis-related proteins. Soy isolate hydrolysate decreased fat accumulation and blood lipid profile by increasing excretion of fat in Sprague-Dawley rats by Aoyama et al. (80). Protease and pepsin prepared egg white hydrolysates reduced lipid content in liver and muscle along with total body fat in rats (81–83). Egg hydrolysate and black soy hydrolysate effects on diabetes and obesity markers in human individuals need more focussed study for their mechanism of absorption and the molecular targets (84, 85). Anti-appetizing peptides from milk suppress appetite and thus prevent weight gain and obesity. High-protein diets with whey proteins decrease appetite, resulting in less fat deposition and an improvement in insulin sensitivity. Zhang and Beynen (86) reported the effect of whey protein in the diet on lowering the level of LDL cholesterol, and increasing the release of cholecystokinin which is also known as appetite-suppressing hormone. Whey protein was isolated with peptide fractions κ-casein f, a glycomacropeptide that is manufactured by Davisco, USA (87). These peptides aid weight management due to the satiety inducing glycomarcopeptide and opioid peptides (7). The bioactivity of total whey protein may be dependent on the sequences of the peptides and its combination with active whey protein fractions (88). A peptide ValProPro derived from milk inhibited obesity-induced adipose tissue inflammation under the cascading effect of angiotensin-converting enzyme (ACE). Thus, ValProPro has been suggested as a viable therapeutic choice for obesity-associated adipose tissue inflammation and insulin resistance (89). Camel milk peptides displayed novel antidiabetic and anti-obesity peptides (90). A pentapeptide GluGlnArgProArg from rice bran < 5 kDa fraction showed around 70% adipocyte viability and a protective role against obesity (91). The antiobesity food peptides with their mechanism of action are listed in Table 2.

**Table 2:** Peptidomimetics and synthetic peptides for antiobesity effects.

**Mimetic**	**Mechanism of action**	**References**
Apolipoprotein A-I mimetic peptides synthesized from D -amino acids (D4F) Ac-AspTrp PheLysAlaPheTyrAspLysValAlaGluLysPheLysGluAlaPhe-NH2	increase in energy expenditure and up-regulation of UCP1 in brown fat	(9, 52–54)
N-terminally acetylated and C-terminally amidated L Ac- AspTrpPheLysAlaPheTyrAspLysValAlaGluLysPheLysGluAlaPhe-NH2	decrease in visceral fat increase in adiponectin, pAMPK, pAKT	(92)
ApoB100-mimetic B4T ([His tag]-[B1-linker]4-T, 147 amino acids)	prevented HFD-induced body weight increases	(56)
H-CysLysGlyGlyArgAlaLysAspCysGlyGlyAsp(LysLeuAlaLysLeuAlaLys)2	kill fat cells, decrease the volume and mass of the subcutaneous fat	Ablaris/MD Anderson- Phase 1 clinical trial http://ir.arrowheadpharma.com/static-files/7b9826c9-9bc7-4497-b7cf-b6ce31b66b9d
BL-3020 (PheDPheArgTrpGly)	binds to receptors in the brain that controls appetite	BioLineRx- Discontinued preclinical trials in Obesity in Israel; (93)
JMV-2959 (1,2,4-triazole ghrelin receptor antagonist)	GHS-R1a receptor antagonist	Aeterna Zentaris- Removed due to unknown reasons, although extensive preclinical research was successful (94)

## Marine Peptides

Marine peptides help aid weight maintenance by reducing the post-prandial blood sugar levels (95, 96). Recently, Fan et al. (97) reported the anti-obesity properties of *Spirulina platensis*-derived peptides which has attracted the focus once again to marine originated peptides. Chitosan, a polysaccharide is already known to modulate the level of serum leptin, C- reactive protein and inhibits the adipocyte differentiation in obese rats (98, 99). Fucoxanthin, alginates, fucoidans, and phlorotannins from seaweeds have been covered well and reviewed for their anti-obesity potential (100). Some marine peptides resemble gastrin, cholecystokinin and/or stimulate their release resulting in appetite control. Small peptides extracted from shrimp stimulate the release of cholecystokinin in STC-1 cells (101). Biofunctional peptides and their mining can be done from fish and shellfish waste components (102). Seaweed *Plocamium telfairiae* and *Spirulina platensis* exhibit the highest lipogenesis inhibitory effect in 3T3-L1 cells (97, 103). Four novel peptides AsnAlaLeuLysCysCysHisSerCysProAla, LeuAsnAsnProSerValCysAspCysAspCysMetMetLysAlaAlaArg, AsnProValTrpLysArgLys, and CysAlaAsnProHisGluLeuProAsnLys slowed down 3T3-L1 cells proliferation (32.29–60.08%). Intake of cod decreased the adipose tissue mass and hepatic lipids in mice (104). AspIleValAspLysIleGluIle peptide from boiled tuna inhibited CCAAT/enhancer-binding proteins (C/EBPs) and expression of peroxisome proliferator-activated receptor gamma (PPAR-γ). It also caused activation of the Wnt/β-catenin pathway, which inhibited pre-adipocytes differentiation into fat globule cells (105). In another work, Henda et al. (106) reported small peptides AlaPro, ValAlaPro, and AlaLysLys, and studied them for their effect on the viability of adipocytes during the proliferation period, among many LysTrp and ValTrp affected the viability during the differentiation stage. Peptides GlyProLeu and IleTyr decreased the final lipid content, glycerol-3-phosphate dehydrogenase activity and the mRNA level of adipocyte markers along with down regulation of two key regulators of adipogenesis—PPARγ and C/EBPα expression. Marine collagen peptide (MCPs) reduced the levels of total triglycerides, total cholesterol, low-density lipoprotein, free-fatty acids along with fasting blood glucose, fasting blood insulin and human glycated hemoglobin A1C in Chinese diabetic patients (107). MCPs in the size range of 2–26 kDa decreased body weight in animal models (108). Fish collagen peptide from a water-hydrolyzed fraction decreased the accumulation of lipid and also decreased expression of C/EBPα, PPARγ, and adipocyte protein 2 (aP2) genes during the 3T3-L1 preadipocytes differentiation (109). Oral supplementation of collagen fragments decreased body fat and weight with an improved cytokine (110). Recent studies have shown anti-oxidant potential of food peptides. Hu et al. (111) isolated novel peptides from Pecan protein hydrolysate and a peptide with amino acid sequence LeuAlaTyrLeuGlnTyrThrAspPheGluThrArg (mol. wt. < 3 kDa) which exhibited strongest antioxidant activity. This novel peptide is suggested to be further evaluated for the possibility of its development as a functional food with pharmaceutical properties. A smaller molecular weight peptide from carrot seed protein also showed strong antioxidant activity (112). Marine functional bioactives and ingredients in bakery and pasta products are also currently projected for future applications (113). All other antiobesity marine peptides are listed in Table 3.

**Table 3:** Peptidomimetics and peptides from food and marine sources for antiobesity effects.

**Soy protein isolate-** IleLeuLeu, LeuLeuLeu, ValHisValVal	Lipolysis of adipose tissue	(114)
**Black Soybean Peptides supplement-** AsnLeuGlnGlyGluAsnGluGluGluAspSerGlyAlaIleValThrValLys, ValSerIleIleAspThrAsnSerLeuGluAsnGlnLeuAspGlnMetProArg, LysGluGlnGlnGlnGluGlnGlnGlnGluGluGlnProLeuGluValArg, GluGlnGlnGlnGluGlnGlnGlnGluGluGlnProLeuGluValArg, GlyAsnProAspIleGluHisProGluThrMet, LeuAspThrSerAsnPheAsnAsnGlnLeuAspGlnThrProArgValPhe, AsnGlnGluGlnGluPheLeuLysTyrGln, ArgLeuLeuLeuLeuLeuGlyTrpLeuLeuIleIleValGlyValIleLeuLeuValGlySerThrLys, LysGluGlnGlnGlnGluGluGlnGlnGluGluGlnProLeuGluValArg, IleIleAspThrAsnSerLeuGluAsnGlnLeuAspGlnMetProArg, LeuAspThrSerAsnPheAsnAsnGlnLeuAspGlnAsnProArgValPhe, GluGlnGlnGlnArgGlnGlnGlnGluGluGlnProLeuGlu, and ProMetAspTyrTyrSerAspTyrAspAspAsnAlaAspAspTyrPheAspAspAlaAsp AspSerAspArg	Causes favorable changes in the metabolites. (unknown mechanism)	(115)
**Peptides from purified soybean β-conglycinin-** LysAsnProGlnLeuArg, GluIleThrProGluLysAsnProGlnLeuArg and ArgLysGlnGluGluAspGluAspGluGluGlnGlnArgGlu	The soy peptides GluIleThrProGluLysAsnProGlnLeuArg and ArgLysGlnGluGluAspGluAspGluGluGlnGlnArgGlu reduced the de novo fatty acid synthesis.	(116)
**Peptide derived from flavourzyme-soy proteinisolate hydrolysate-** ValHisValVal	VHVV suppressed TNF-α expression. It increased Bcl-2 expression in and suppressed Bad expression, suggesting a cytoprotective effect conferred on skeletal muscles. Act as antiapoptotic by decreasing the levels of other Caspase 3 cleaved caspase 9 and cytochrome c in all the doses tested. It regulated the levels of PPAR-α which were suppressed in obese mice.	(79)
**Isoflavone-free peptide mixture (BSP) from black soybean-** AsxSerProIleProProGlyValProTyr	The phosphorylation of AMPK was activated but with phosphorylation inhibition of ACC, indicating its hypotriglyceridemic effect.	(78)
**Enzymatic hydrolysates of β-conglycinin-** ValArgIleArgLeuLeuGlnArgPheAsnLysArgSer	It stimulates CCK release as an exogenous CCK releasing peptide, which results into suppression of appetite via CCK-A receptors.	(117)
**Hydrophilic fractions from soy crude peptide** LysAla ValLys SerTyr	Reduces TG synthesis	(118)
**Soy glycinin peptides-** IleAlaValProGlyGluValAla, IleAlaValProThrGlyValAla, and LeuProTyrPro	Cholesterol-lowering activity by activating the LDLR-SREBP2 pathway and the activation of AMPK and ERK 1/2	(119)
*Thunnus. thynnus, T. albacares, T. alalunga, and T. obesus* Beta-enolase, 47.5 kDa		(120)
**Black soy peptides-** AsnLeuGlnGlyGluAsnGluGluGluAspSerGlyAlaIleValThrValLys, ValSerIleIleAspThrAsnSerLeuGluAsnGlnLeuAspGlnMetProArg, LysGluGlnGlnGlnGluGlnGlnGlnGluGluGlnProLeuGluValArg, GluGlnGlnGlnGluGlnGlnGlnGluGluGlnProLeuGluValArg, GlyAsnProAspIleGluHisProGluThrMet, LeuAspThrSerAsnPheAsnAsnGlnLeuAspGlnThrProArgValPhe, AsnGlnGluGlnGluPheLeuLysTyrGln, ArgLeuLeuLeuLeuLeuGlyTrpLeuLeuIleIleValGlyValIleLeuLeuValGlySerThrLys, LysGluGlnGlnGlnGluGluGlnGlnGluGluGlnProLeuGluValArg, IleIleAspThrAsnSerLeuGluAsnGlnLeuAspGlnMetProArg, LeuAspThrSerAsnPheAsnAsnGlnLeuAspGlnAsnProArgValPhe, GluGlnGlnGlnArgGlnGlnGlnGluGluGlnProLeuGlu and ProMetAspTyrTyrSerAspTyrAspAspAsnAlaAspAspTyrPheAspAspAlaAsp AspSerAspArg		(115)
**Desalinated boiled tuna-** AspIleValAspLysIleGluIle		(121)
**Defatted oat meal hydrolysate-** PheLeuGlnProAsnLeuAspGluHis, AspLeuGluLeuGlnAsnAsnValPheProHis and ThrProAsnAlaGlyValSerGlyAlaAlaAlaGlyAlaGlyAlaGlyGlyLysHis		(122)
**Fragment of soybean β-conglycinin** (position 53–61)- ValArgIleArgLeuLeuGlnArgPheAsnLysArgSer		(117)
**Soy protein isolate-** LysAla, ValLys and SerTyr		(118)
**Fermented protein hydrolysates from sardinelle** (*Sardinella aurita*)—peptides in the range of 150–900 Da.		(123)
**Indian major carp, rohu** (*Labeo rohita* Ham.)- Rohu leptin is 16283.38 Da		(124)

## Conclusion and Future directions

Peptides have been known for several decades from different sources with promising activity but very few studies have been carried out *in vivo*. Initially they were isolated from live animals and now they hold a specific therapeutic potential in the pharmaceutical field. They have been widely tested in new regimens due to rapid development in molecular biology *in vitro* assays. Newer approaches in chemistry are helping to improve the peptide stability and its pharmaceutical property. Due to their various limitations, continued study of natural peptides from bioresources with newer strategies of peptide engineering and their mimetics is suggested to be explored extensively. The number of therapeutic peptides and mimetics will increase substantially if advances in peptide engineering, combined uses of peptides, dual or triple agonists, aptamers, cell-penetrating, and multifunctional peptides are utilized to their highest potential. The current shortcomings faced by peptide drugs can be addressed by developing a deeper understanding of natural peptides from food and marine sources. Computational biology currently is a great support for peptide drug discovery and omics approaches uses genomics, proteomics, transcriptomics, and metabolomics as modern age platforms for finding bioactive peptides with unique structural features. Any novel finding pertaining to peptides are capable of shifting all the concepts toward obesity targets, they supported by evidence based studies and hence hold more promising future to control body weight. A new peptide, mimetic, and analog can be further taken for a development of suitable drug delivery system, stable formulation, with increased half-life to be used as an antiobesity drug. Though there is an increased interest in peptides and their mimetics, more efforts are required to meet the criteria to develop them as a therapeutic drug. Further studies of peptide's structural scaffold, their functions and mechanism of action, and other mediators involved in obesity can help discovery and development of novel peptides.

## Author Contributions

MK performed the initial literature research and wrote the structured manuscript, corrected and prepared the final version of the manuscript.

### Conflict of Interest Statement

The author declares that the research was conducted in the absence of any commercial or financial relationships that could be construed as a potential conflict of interest.
